# Timing and Impact of Psychiatric, Cognitive, and Motor Abnormalities in Huntington Disease

**DOI:** 10.1212/WNL.0000000000011893

**Published:** 2021-05-11

**Authors:** Branduff McAllister, James F. Gusella, G. Bernhard Landwehrmeyer, Jong-Min Lee, Marcy E. MacDonald, Michael Orth, Anne E. Rosser, Nigel M. Williams, Peter Holmans, Lesley Jones, Thomas H. Massey, on behalf of the REGISTRY Investigators of the European Huntington's Disease Network

**Affiliations:** From the Division of Psychological Medicine and Clinical Neurosciences (B.M., N.M.W., P.H., L.J., T.H.M.), Brain Repair Group (A.E.R.), Schools of Medicine and Biosciences, and Neuroscience and Mental Health Research Institute (A.E.R.), Cardiff University, UK; Molecular Neurogenetic Unit (J.F.G., J.-M.L., M.E.M.), Center for Genomic Medicine, Massachusetts General Hospital; Department of Genetics (J.F.G., J.-M.L., M.E.M.), Harvard Medical School, Boston, MA; Department of Neurology (G.B.L.), University of Ulm, Germany; and Swiss Huntington's Disease Centre (M.O.), Siloah, Bern, Switzerland.

## Abstract

**Objective:**

To assess the prevalence, timing, and functional impact of psychiatric, cognitive, and motor abnormalities in Huntington disease (HD) gene carriers, we analyzed retrospective clinical data from individuals with manifest HD.

**Methods:**

Clinical features of patients with HD were analyzed for 6,316 individuals in an observational study of the European Huntington's Disease Network (REGISTRY) from 161 sites across 17 countries. Data came from clinical history and the patient-completed Clinical Characteristics Questionnaire that assessed 8 symptoms: motor, cognitive, apathy, depression, perseverative/obsessive behavior, irritability, violent/aggressive behavior, and psychosis. Multiple logistic regression was used to analyze relationships between symptoms and functional outcomes.

**Results:**

The initial manifestation of HD is increasingly likely to be motor and less likely to be psychiatric as age at presentation increases and is independent of pathogenic CAG repeat length. The Clinical Characteristics Questionnaire captures data on nonmotor symptom prevalence that correlate specifically with validated clinical measures. Psychiatric and cognitive symptoms are common in HD gene carriers, with earlier onsets associated with longer CAG repeats. Of patients with HD, 42.4% reported at least 1 psychiatric or cognitive symptom before motor symptoms, with depression most common. Each nonmotor symptom was associated with significantly reduced total functional capacity scores.

**Conclusions:**

Psychiatric and cognitive symptoms are common and functionally debilitating in HD gene carriers. They require recognition and targeting with clinical outcome measures and treatments. However, because it is impossible to distinguish confidently between nonmotor symptoms arising from HD and primary psychiatric disorders, particularly in younger premanifest patients, nonmotor symptoms should not be used to make a clinical diagnosis of HD.

**Trial Registration Information:**

ClinicalTrials.gov Identifier: NCT01590589

Huntington disease (HD) is a central neurodegenerative disorder caused by an expanded CAG repeat (>35 CAGs) in the *Huntingtin* gene.^1^ Longer repeats are associated with earlier disease onset.^2,3^ Neuronal loss in the brain causes progressive motor abnormalities, cognitive decline, and ultimately death. The movement disorder usually includes chorea but may also involve dystonia, ataxia, oculomotor problems, and parkinsonism, some of which are initially identifiable only through targeted HD examination. Debilitating behavioral and psychiatric symptoms are common in HD gene carriers and require treatment, although they cannot be used in clinical practice to define HD onset because it is impossible to distinguish psychiatric manifestations of HD from coincident diagnoses.^4,5^ Prospective studies of HD gene carriers many years from predicted clinical onset have shown only subtle motor, cognitive, and psychiatric deficits compared with age- and sex-matched controls.^6-8^ This implies that there is a window for therapeutic intervention to preserve normal brain functions. Understanding in detail the timing and impact of different symptoms in HD gene carriers will help improve targeted therapies.

The HD Clinical Characteristics Questionnaire (HD-CCQ)^9^ gathers retrospective data from individuals with HD about the prevalence and timing of 8 motor, cognitive, and psychiatric symptoms.^10^ Here, we validate HD-CCQ data for nonmotor symptoms by showing strong and specific associations with established scores of depression, irritability, and cognition. We use HD-CCQ data to show the high prevalence of psychiatric and cognitive symptoms in HD gene carriers, often in advance of motor symptoms, and their negative impact on the lives of patients.

## Methods

### Standard Protocol Approvals, Registrations, and Patient Consents

Participants were in the multicenter, multinational An Observational Study of the European Huntington's Disease Network (REGISTRY) study of European HD (ehdn.org/wp-content/uploads/2018/06/registry-protocol-3.0.pdf; NCT01590589). Data were accessed as part of European Huntington's Disease Network (EHDN) data mining project 0791. Ethics approval for REGISTRY was obtained in each participating country. All participants gave written informed consent.

### Participant Data

HD participant data, collected from June 2004 to February 2016 across 161 sites in 17 European countries, were obtained for 6,316 individuals (accessed October 2016) who had clinical HD onset, determined by the rating clinician in REGISTRY, and a confirmed pathogenic CAG length of 36 to 93. Of these CAG sizes, 5,027 were centrally determined by BioRep Inc (Milan, Italy; REGISTRY protocols), and 1,289 were derived by local diagnostic laboratories. Two estimates of the age at onset of symptoms or signs in HD were used in this study. First, the clinician-estimated age at first HD manifestation was based on all available clinical evidence at the first REGISTRY visit (coded as sxrater). Having an sxrater age at onset was required for inclusion in this study. Onset type was classified as motor, cognitive, psychiatric, oculomotor, other, or mixed. Because the clinician's estimate was given as a date, age estimates were calculated from the participant's anonymized birthday; when only a year was given, July 15 was used for estimation (15/07/xxxx). Second, the ages at onset of different symptoms in patients with HD were estimated by the HD-CCQ, which was completed by a health care professional, usually an HD-specialist nurse or similarly qualified person, using responses from the individual with HD and their care partners (present in clinic in 93.1% of cases) and patient medical notes. The HD-CCQ comprises questions about 8 symptoms commonly observed in HD, asking whether the participant has ever had the symptom (yes or no) and, if yes, the age at which the symptom was first experienced (appendix 3, doi.org/10.5061/dryad.pk0p2ngkz). Information was available, at least in part, for 5,609 individuals. The symptoms recorded (number of individuals with data) were as follows: motor (chorea or other, consistent with HD) 5,603; cognitive impairment sufficient to affect work or daily living 5,591; apathy 5,584; depression 5,595; perseverative/obsessive behavior 5,588; irritability 5,586; violent or aggressive behavior 5,586; and psychosis 5,589. For subsequent analyses, missing data were handled using pairwise deletion to maximize the number of individuals. Typically, the rater estimate of clinical onset and initial HD-CCQ would be recorded at the first REGISTRY visit, sometimes by 1 clinician and sometimes by a clinician and another qualified staff member such as HD-specialist nurse, depending on local clinic setup. Subsequent visits updated the HD-CCQ; we used data from the most recent clinic visit. We had data on Shoulson-Fahn disease stage at last clinic visit for 4,554 individuals (72.1% of our study population): stage 1 (total functional capacity [TFC] 11–13; n = 890, 19.5%), stage 2 (TFC 7–10; n = 1,278, 28.1%), stage 3 (TFC 4–6; n = 969, 21.3%), stage 4 (TFC 1–3; n = 1,133, 24.9%), and stage 5 (TFC 0; n = 284, 6.2%).

The Hospital Anxiety/Depression Scale (HADS) and Snaith Irritability Scale (SIS) were completed by the participant at each clinic visit and provide measures of anxiety, depression, and irritability at that specific time. We used lifetime highest total depression and total irritability scores from both the HADS and the SIS in analyses. Similarly, the Symbol-Digit Modalities Test (SDMT) and Stroop tests of cognitive ability were administered as part of the Unified Huntington's Disease Rating Scale (UHDRS)^11^ at each visit. The UHDRS consists of validated questionnaires, tools, and examinations related to motor, cognitive, behavioral, and functional impairments seen in HD. For the SDMT and Stroop tests, we used the total correct scores from the most recent clinic visit. Disease duration was estimated by taking the most recent visit and subtracting the clinician's estimate of disease onset. The product of short form of the Problem Behaviours Assessment (PBA-s) severity and frequency scores from the most recent clinic was used for modeling purposes.

### Statistical Analyses of Clinical Data

Total depression scores from the HADS, total irritability scores from the SIS, the number of correct answers on the SDMT, the number of correct answers on Stroop tests, or composite PBA-s scores were regressed on HD clinical characteristics data, age, CAG length, sex, and disease duration (table 1). To calculate coefficients of determination (*R*^2^ values, table 2), HD-CCQ age at onset data were natural log transformed. Only individuals with a known sex and a symptom onset ≥3 years were considered, and a residual vs leverage plot identified 1 influential data point passing the Cook distance that was removed from all *R*^2^ calculations. The *p* values were calculated comparing male and female *R*^2^ values with the Fisher transformation.^12^ A χ^2^ test was used to test for differences in symptom frequency, derived from the yes/no component of the HD-CCQ, between male and female participants.

**Table 1:**
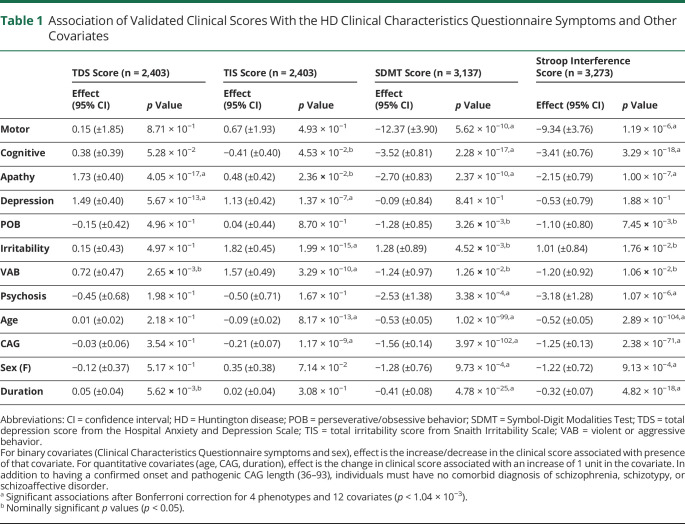
Association of Validated Clinical Scores With the HD Clinical Characteristics Questionnaire Symptoms and Other Covariates

**Table 2:**
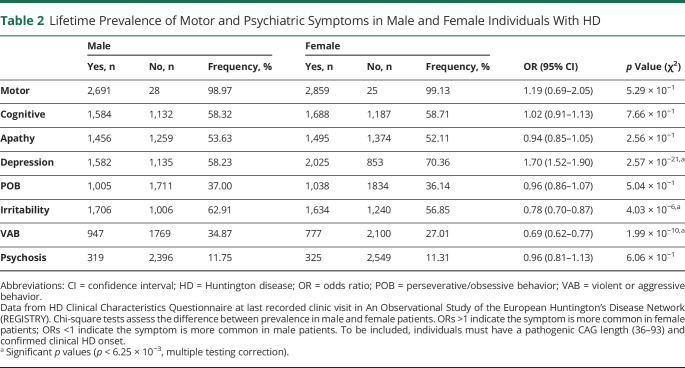
Lifetime Prevalence of Motor and Psychiatric Symptoms in Male and Female Individuals With HD

**Figure 1:**
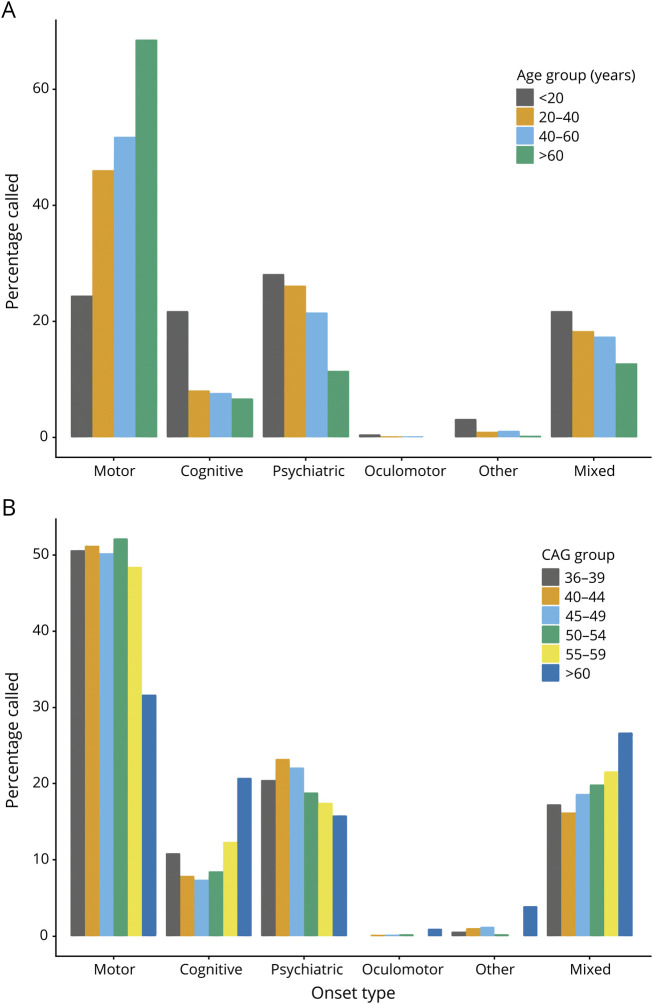
Initial Manifestation of HD Varies With Age and CAG Length All included individuals had a pathogenic CAG length (36–93) and confirmed Huntington disease (HD) onset age determined by a rating clinician. (A) Frequency of different onset types in 4 age groups, chosen to show juvenile HD and then 20-year bins for clarity. Total n= 6,289, <20 years n = 188, 20 to 40 years n = 2,216, 40 to 60 years n = 3,276, >60 years n = 609. (B) Frequency of different onset types in 6 CAG length groups, chosen for clarity across the pathogenic range. Total n = 6,289, 36 to 39 CAG n = 156, 40 to 44 CAG n = 3,813, 45 to 49 CAG n = 1,735, 50 to 54 CAG n = 387, 55 to 59 CAG n = 97, >60 CAG n = 101.

Associations between binary responses in the HD-CCQ (1 = experienced the symptom, 0 = symptom not experienced) and clinical covariates were tested with logistic regression. The covariates used were sex, CAG length, alcohol consumption (units per week), tobacco use (cigarettes per day), education (years of education), TFC score, and total motor score (TMS). An additional analysis regressed the type of HD onset defined by the clinician, coded as a binary variable, on the clinician's onset or CAG length (table e-2, doi.org/10.5061/dryad.pk0p2ngkz). This analysis was restricted to participants with HD with 36 to 59 CAGs to be consistent with figure 1 subgroups and to individuals with adult-onset HD (≥20 years). We also tested whether symptom presence was associated with the length of the wild-type (6–35 CAGs) and expanded (CAG repeat length of 36–93) CAG alleles in individuals of known sex and for whom both CAG lengths were known (table e-3, doi.org/10.5061/dryad.pk0p2ngkz). Nineteen individuals with a coincident formal diagnosis of schizophrenia, schizotypal disorder, or schizoaffective disorder (ICD-10 code F20, F21 or F25) were excluded from all models, although it was not possible to formally exclude these symptoms being part of the HD phenotype. Statistical analysis used R (version 3.6.0; R Core Team, 2019, r-project.org/).

### Data Availability

Further information and data requests should be directed to Thomas H. Massey (MasseyT1@cardiff.ac.uk). Anonymized summary data are available to qualified investigators. Furthermore, anonymized patient data are available from the EHDN on request given institutional assurance that patient confidentiality will be upheld and no attempt will be made to discover the identity of patients.

## Results

### The Initial Manifestation of HD Varies With Age and CAG Length

The age at onset of the first unequivocal motor features of HD (motor onset) has been used as a specific milestone in the natural history of HD in individuals, although it is only a crude measure of a progressive neuropathologic process. It has proved particularly useful in recent genetic modifier studies of HD.^13,14^ The first psychiatric and cognitive manifestations of HD are more difficult to define with certainty, being less specific for HD and clinically indistinguishable from common coincident psychiatric diagnoses (e.g., depression), particularly in younger patients many years from predicted motor onset. The timing of the first unequivocal feature of HD is typically recorded retrospectively by a rating physician in observational studies such as REGISTRY according to clinical information and symptom history from patients and care partners.^9,15,16^ The rater also records the initial major presenting feature of a choice of 6: motor, cognitive, psychiatric, oculomotor, other, or mixed. We analyzed the initial manifestation of HD for 6,316 participants in REGISTRY,^9^ including 3,083 male (48.8%) and 3,233 female (51.2%) participants. All participants had a confirmed genetic diagnosis of HD with a pathogenic CAG repeat length of 36 to 93 (figure e-1, doi.org/10.5061/dryad.pk0p2ngkz). The first manifestation of HD, determined by the rating physician, varied with patient age (figure 1A and table e-1, doi.org/10.5061/dryad.pk0p2ngkz). Individuals with onset before 20 years of age, defined as juvenile HD, were equally likely to present with motor (24.5%), cognitive (21.8%), or psychiatric features (28.2%). In contrast, the initial manifestation of HD was more likely to be motor than psychiatric in adult-onset HD. As age at the first manifestation increased (figure 1A and table e-2A, doi.org/10.5061/dryad.pk0p2ngkz), motor presentations became more likely (odds ratio [OR] 1.06 per 10-year increase in onset age, 95% confidence interval [CI] 1.04–1.07; *p* = 7.4 × 10^−22^), but psychiatric presentations became less likely (OR 0.96 per 10-year increase in onset age, 95% CI 0.95–0.97; *p* = 9.4 × 10^−16^). For people presenting at >60 years of age, more than two-thirds (68.6%) had initial motor abnormalities, with far fewer having psychiatric (11.5%) or cognitive (6.7%) presentations. Next, we tested whether there was any relationship between pathogenic CAG repeat length, known to be inversely correlated with age at clinical onset, and the presenting phenotype. There was no significant relationship between CAG length (36–59 inclusive) and the relative proportions of motor, cognitive, and psychiatric onset cases (figure 1B and table e-2B, doi.org/10.5061/dryad.pk0p2ngkz). For the few cases with data and repeat lengths of >59 CAGs, we observed a more balanced distribution of motor, cognitive, and psychiatric presentations, mirroring the trends seen for the cases of juvenile HD.

### Psychiatric and Cognitive Symptoms Captured by HD-CCQ Correlate With Scores From Validated Clinical Tools

The HD-CCQ was introduced to later versions of REGISTRY as the best retrospective way of capturing symptom data in existing HD populations. It is completed by a health care professional using information from individuals with HD and their care partners, present in clinic for >93%, about lifetime history and age at onset of 8 symptoms typical of HD. These symptoms are motor (compatible with HD), depression, irritability, violent or aggressive behavior, apathy, perseverative/obsessive behavior, psychosis, and cognitive impairment sufficient to affect work or daily living. In REGISTRY, this information was updated at each annual clinic visit. In HD-CCQ, motor symptoms are not specified beyond being compatible with HD, limiting the utility of motor data, but psychiatric and behavioral symptoms are clearly defined.

Because prevalence data from HD-CCQ have not been used in large analyses before, we first tested how well they correlated with validated clinical scores of depression (HADS), irritability (SIS), and cognition (SDMT and Stroop). To mitigate against potential effects of medication at certain times, we used the lifetime highest total depression and total irritability scores for each individual. For cognitive tests, we used scores at the last recorded clinic visit because these would be expected to worsen progressively and to be little affected by medication. Total depression score from HADS was significantly increased in individuals with depression recorded in HD-CCQ (increase of 1.49 units, 95% CI 1.09–1.89; *p* = 5.7 × 10^−13^; table 1). An increase in HADS score was also observed in individuals with HD-CCQ apathy, probably because apathy, common in HD, may be mistaken for depression by individuals and their care partners when completing the HD-CCQ. Total irritability score from SIS was significantly increased in individuals with HD-CCQ irritability (increase of 1.82 units, 95% CI 1.37–2.27; *p* = 2.0 × 10^−15^) and with violent/aggressive behavior (increase of 1.57 units, 95% CI 1.08–2.06; *p* = 3.3 × 10^−10^), as expected. Both SDMT and Stroop scores of cognitive ability were significantly decreased in individuals with cognitive impairment as recorded in HD-CCQ (reductions of 3.52 units, 95% CI 2.71–4.33; *p* = 2.3 × 10^−17^ and 3.41 units, 95% CI 2.65–4.17; *p* = 1.4 × 10^−22^, respectively). Significant associations between cognitive scores and motor and apathy symptoms were also observed. In addition, we found robust and specific associations between neuropsychiatric symptoms recorded in HD-CCQ and their related symptoms scored with the validated PBA-s (supplemental table e-4, doi.org/10.5061/dryad.pk0p2ngkz). The specificity of the associations between HD-CCQ data and recognized clinical scales validated the use of HD-CCQ data in subsequent analyses.

### Psychiatric Symptoms Are Common in HD Gene Carriers and Are Associated With CAG Repeat Length

We next analyzed the lifetime prevalence of the 8 symptoms recorded in HD-CCQ in 5,609 individuals with HD at their most recent clinic visit (table 2). The mean age at last recorded clinic visit was 53.3 years: 53.5 years for male participants with data (range 10.4–92.6 years; n = 2,569) and 53.2 years for female participants (range 7.9–90.2 years; n = 2,698). Almost all (>99%) had experienced motor symptoms compatible with HD, indicating why motor abnormalities remain the diagnostic standard for clinical onset of HD. Although motor symptoms are not defined explicitly in HD-CCQ, contemporaneous data from UHDRS showed that 96.8% of our study population had chorea, along with variable amounts of incoordination, dystonia, and rigidity. In HD gene carriers, these motor symptoms are likely to be specific manifestations of HD. The next most prevalent symptom was depression, occurring in 64.5% of individuals with HD, with significantly more female patients affected than male patients (70.4% vs 58.2%; OR 1.70, 95% CI 1.52–1.90; *p* = 2.6 × 10^−21^). Cognitive impairment sufficient to affect work or activities of daily living, apathy, and irritability were also each observed in more than half of our HD population. Cognitive impairment and apathy were equally likely in male and female participants, but significantly more irritability was observed in male participants (62.9% vs 56.9%, OR 0.78, 95% CI 0.70–0.87; *p* = 4.0 × 10^−6^). An excess of violent or aggressive behavior was also observed in the male group (34.9% vs 27.0%, OR 0.69, 95% CI 0.62–0.77; *p* = 2.0 × 10^−10^). Psychosis was the least prevalent of the 8 recorded symptoms, although this was still observed in >11% of individuals with HD with no significant difference in prevalence between male and female participants.

**Figure 2:**
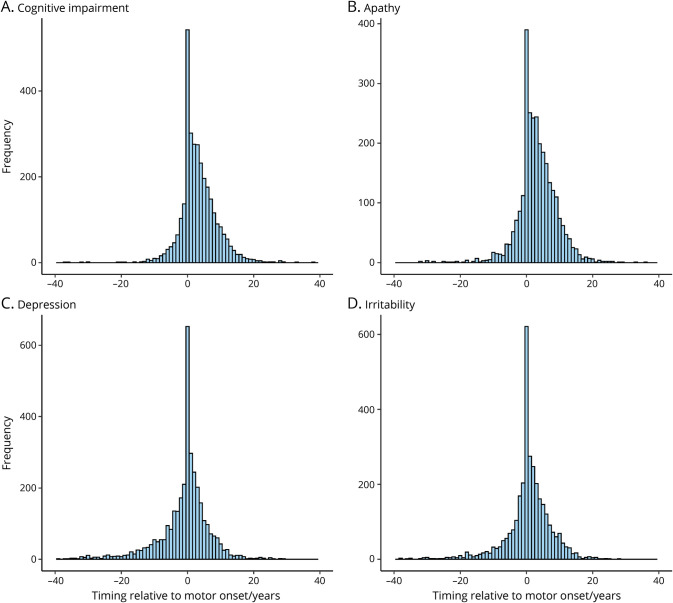
Onsets of Cognitive and Psychiatric Symptoms Relative to Motor Onset in HD Age at onset of motor symptoms was subtracted from the age at onset of each cognitive/psychiatric symptom when present. Timings of up to ± 40 years relative to motor onset shown. Only individuals with a rater-confirmed age at onset and CAG length (36–93) were included. Data from Huntington Disease (HD) Clinical Characteristics Questionnaire. (A) Cognitive impairment n = 3,225; (B) apathy n = 2,852; (C) depression n = 3,495; and (D) irritability n = 3,235.

There was a strong inverse correlation between pathogenic CAG repeat length (40–55 CAG inclusive) and mean age at symptom onset for all symptoms analyzed (figure 2). We found no effect of wild-type CAG allele length on any symptom onset and no any significant statistical interaction between expanded and wild-type repeat lengths (table e-3, doi.org/10.5061/dryad.pk0p2ngkz). Pathogenic CAG length explained 66.3% of the variance in age at onset of motor symptoms, in line with previous estimates,^2,3,17-23^ but also between 37.5% and 61.9% of the variance in onset of each of the psychiatric symptoms analyzed (table 2). Depression had the weakest association with CAG repeat length (*R*^2^ = 37.5%). CAG length accounted for significantly more of the variance in age at onset of perseverative/obsessive behavior in male participants (*p* = 3.7 × 10^−3^; table 2) and irritability in female participants (*p* = 1.3 × 10^−3^).

### Timing of Motor and Psychiatric Symptoms in HD Gene Carriers Varies With Symptom Type and CAG Length

Given that motor onset is often used as a specific milestone in the natural history of HD, we investigated the timing of each of the 7 psychiatric/cognitive symptoms relative to the age at first motor symptoms recorded in HD-CCQ (figure 2). The differences in ages between first motor symptoms and each of the psychiatric symptoms were approximately normally distributed, with a wide range of at least ±20 years in each case (figure 2 and figure e-2, doi.org/10.5061/dryad.pk0p2ngkz). In those patients reporting depression, onset occurred before motor symptoms in 39.2% (n = 1,369 of 3,495). For patients with irritability, onset occurred before motor symptoms in 30.8% (n = 996 of 3,235). Perseverative/obsessive behavior tended to occur later in the disease course, after motor symptoms, as did psychosis, although numbers were smaller. Cognitive impairment and apathy had the most positively skewed distributions, with onset occurring after motor onset in 2,179 of 3,225 (67.6%) and 1,981 of 2,852 (69.5%) of individuals, respectively. Overall, 42.4% of patients with HD (n = 2,140 of 5,042) reported at least 1 psychiatric or cognitive symptom in advance of motor symptoms, with a further 22.3% (n = 1,126 of 5,042) reporting at least 1 of these symptoms at the same time as motor abnormalities.

**Figure 3:**
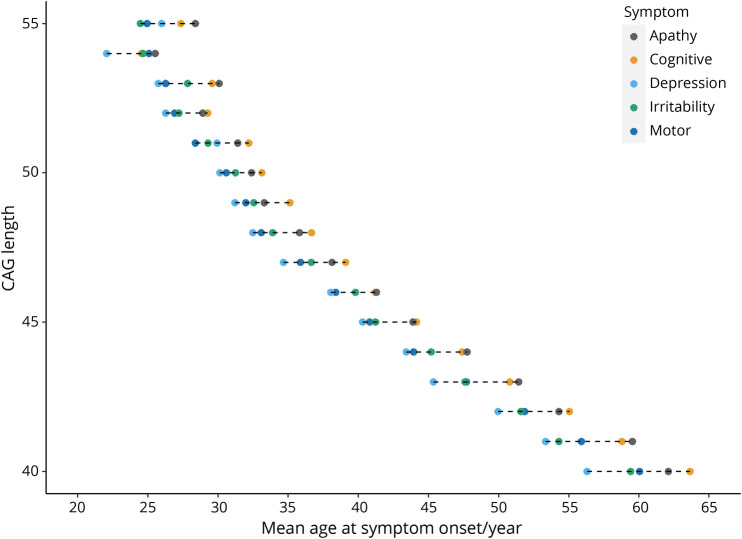
Mean Ages at Onset for Motor and Psychiatric Symptoms at Different CAG Repeat Lengths Shown are the mean ages at symptom onset as recorded by the Huntington Disease Clinical Characteristics Questionnaire for apathy (n = 2,739), cognitive impairment (n = 3,069), depression (n = 3,399), irritability (n = 3,117), and motor symptoms (n = 4,889).

We next assessed whether there were any patterns in the mean ages at onset of the different symptoms when plotted by CAG repeat length (figure 3). Some consistent relationships between symptoms were observed. Depression usually had the youngest mean age at onset, followed by motor impairment and then apathy and cognitive impairment as the latest symptoms. Mean age at onset of irritability preceded that of motor onset at shorter repeat lengths (40–43 CAGs, inclusive) but tended to follow it at longer repeat lengths (44–53 CAGs, inclusive). The mean difference in years from onset of first symptom to last decreased with CAG repeat length from ≈8 years for 40 repeats to 4 years for 55 repeats (figure 3).

### Cognitive and Psychiatric Symptoms Are Significantly Associated With Reduced Functional Capacity

To assess whether psychiatric, cognitive, or motor symptoms were associated with altered functional abilities, we used multiple logistic regression (table 4). This analysis incorporated sex, pathogenic CAG length, duration of disease from clinical onset to last clinic visit, alcohol consumption, tobacco use, educational attainment, TFC score, and TMS as predictors of the presence/absence of each HD-CCQ symptom. The presence of any of the psychiatric or cognitive symptoms was significantly associated with lower TFC, an indication of impaired ability to work, manage personal finances, and function independently. Cognitive impairment was most significantly associated with reduced TFC (OR per 1-unit decrease in TFC 1.28, 95% CI 1.23–1.35; *p* = 1.6 × 10^−25^). Depression was significantly associated with lower TMSs (indicating fewer motor symptoms or signs), fitting with its prevalence early in the disease course. Finally, significant associations were observed between depression and female sex (OR 1.77, 95% CI 1.44–2.17; *p* = 7.0 × 10^−8^) and tobacco use and irritability (OR per 1 extra cigarette per day 1.02, 95% CI 1.01–1.03; *p* = 1.0 × 10^−4^). Although not reaching strict criteria for significance after correction for multiple tests, associations were also found between male sex and irritability (OR 0.75, 95% CI 0.61–0.92; *p* = 5.4 × 10^−3^) and lower educational attainment and psychosis (OR per 1 extra year of education 0.92, 95% CI 0.88–0.97; *p* = 2.2 × 10^−3^).

**Table 3:**
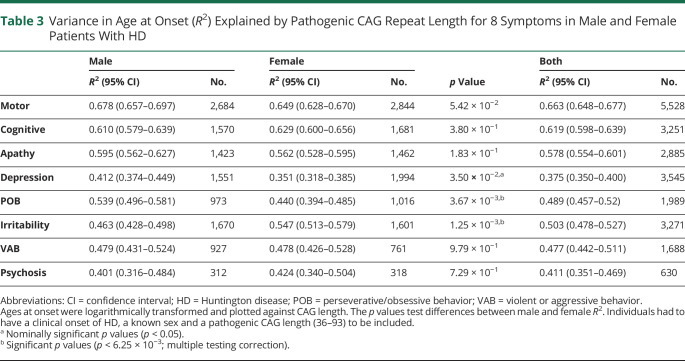
Variance in Age at Onset (*R*^2^) Explained by Pathogenic CAG Repeat Length for 8 Symptoms in Male and Female Patients With HD

## Discussion

In this large study of >6,000 patients, we have shown that the initial manifestation of HD, as determined retrospectively by an expert rater, varies significantly with age. Late presentations (>60 years) are usually associated with motor abnormalities, whereas early presentations (<20 years; juvenile HD) are associated with a wider range of motor, cognitive, and psychiatric abnormalities (figure 1A). These results extend prior studies that have shown that motor presentation of HD is common in late-onset disease (65.5% of an earlier REGISTRY cohort^24^), with more variable presentations in juvenile HD.^25,26^ Approximately 20% of patients with HD present with rater-determined psychiatric features, in line with previous findings (table e-1, doi.org/10.5061/dryad.pk0p2ngkz).^9^ Cognitive onset of HD might be underreported in older age groups because it is regarded as coincident age-related change. Our results show that there is little relationship between pathogenic CAG repeat length and onset type in adult-onset HD (figure 1B), despite both being associated with age at clinical onset. These data fit a model in which age at clinical onset is driven primarily by CAG repeat length but modified by environmental factors and variants at other genomic loci.^14,23,27,28^ The age and physiology of the brain at clinical onset subsequently determine the types of symptoms that become manifest.

The HD-CCQ captures quantitative information not available elsewhere on symptom prevalence and timing in the HD population. Before its introduction in REGISTRY, age at first motor symptoms was not routinely recorded for all patients with HD. HD-CCQ provides particular insight into neuropsychiatric symptoms but is not designed to capture the subtle early motor or cognitive signs found in prospective studies.^7,8^ Because it relies on retrospective reporting by patients and care partners, the HD-CCQ is necessarily coarse, although the data it generates correlate well with more precise measures of depression, irritability, and cognition (table 1). Cognitive impairment measured by SDMT or Stroop tests correlated most strongly with lifetime history of cognitive impairment in HD-CCQ, as expected, but also showed significant correlations with motor symptoms and apathy. These results fit with other studies showing that these symptoms track together in the disease trajectory.^29,30^ There was also a significant association between cognitive impairment and psychosis, which fits the cognitive deficits observed in schizophrenia.^31^ Conversely, validated depression and irritability scores correlated well with their respective prevalence data from HD-CCQ but were not associated with motor or cognitive impairment (table 1).

Almost all patients with HD have specific motor abnormalities consistent with HD during their disease course (table 2). Psychiatric and cognitive symptoms are also very common (table 2), much more prevalent than in non-HD populations,^5,10,32,33^ and likely are underestimated due to pathologic unawareness of these traits by patients with HD.^34^ However, clinically, it is currently impossible to distinguish between symptoms arising as a result of the HD mutation and those arising from primary psychiatric disorders, particularly in younger premanifest patients in whom diseases such as depression are common.^35^ Furthermore, environmental effects on mental health such as living in a family with HD should not be overlooked. Therefore, nonmotor symptoms should not be used to make a clinical diagnosis of HD; doing so could even cause harm in vulnerable individuals with psychiatric symptoms. Future studies of psychiatric and cognitive symptoms and signs in HD gene carriers against gene-negative community controls might help define an HD-specific neuropsychiatric phenotype that would enable more confident attribution of early abnormalities to HD.

The age at onset of each symptom recorded by HD-CCQ was inversely correlated with CAG length (figure 3), with motor symptoms best correlated (table 3). Depression was least correlated (*R*^2^ = 37.5%), likely reflecting the high prevalence of the symptom in the general population independently of HD and the lack of use of universal diagnostic criteria. These data are consistent with previous reports showing that CAG length accounts for 47% to 72% of the variance in age at motor onset of HD^36^ but contradict previous studies that reported no correlation between CAG repeat length and psychiatric symptoms.^37-40^ However, these studies were small and often examined incident psychiatric symptoms, which can fluctuate over time, rather than lifetime history as here. Accurate CAG tract sizing will improve the accuracy of correlations between repeat length and symptoms.^14,41,42^

**Table 4:**
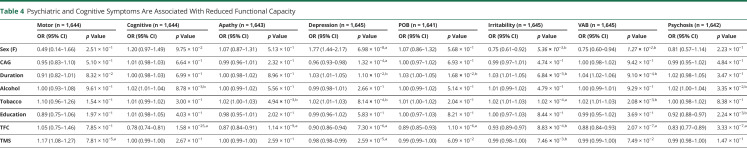
Psychiatric and Cognitive Symptoms Are Associated With Reduced Functional Capacity

Despite considerable variation in the timing of psychiatric and motor symptoms, there are some conserved patterns (figures 2 and 3). Depression and, less often, irritability can precede motor symptoms by many years. Conversely, apathy and cognitive impairment tend to occur after motor symptoms, although patients do recognize and report these symptoms less readily than depression or irritability. Overall, the HD-CCQ data show that 64.8% of our HD population (n = 3,266 of 5,042) reported at least 1 psychiatric or cognitive symptom by the time of the first motor symptoms. This is a much higher figure than previously reported and based on clinician estimates of first HD manifestation (figure 1),^9^ most likely because it is difficult to confidently attribute early psychiatric symptoms to HD. The overlap between HD and psychiatric disorders has been demonstrated by the recent finding that polygenic risk scores for psychiatric diseases, particularly depression and schizophrenia, are associated with increased risk of corresponding psychiatric symptoms in HD.^29^ This suggests that the expanded *HTT* CAG repeat might lower the genetic threshold for manifestation of typical psychiatric symptoms.^29^ In agreement, we found the expected relationships between female sex and depression and male sex and irritability in our cohort (table 4). The nominally significant negative association of psychosis in HD with educational level (table 4) also corroborates work showing that higher levels of education are associated with decreased schizophrenia risk.^43^

We acknowledge several potential limitations of these data. They are retrospective, subject to recall bias, and cross-sectional. Furthermore, HD-CCQ data depend on the interpretation of questions. For example, motor symptoms are not explicitly defined, so although 96.8% of our population had chorea, this was not documented in HD-CCQ. Future iterations might usefully subdivide motor symptoms into (1) fidgety or jerky involuntary movements (chorea) and (2) other HD-related movement problems such as unsteadiness, stiffness, or trouble with fine movements. Our analyses are based on data from the most recent clinic visit, which is at different points of the disease course in different individuals. We controlled for this by using disease duration, the time between first onset and last clinic visit, as a covariate in analyses. The use of psychoactive medications is found in up to 60% of patients with HD and might confound motor and neuropsychiatric phenotypes.^9,44^ Of drugs prescribed for chorea, tetrabenazine can induce depression, and antipsychotics can reduce irritability. They also suppress motor manifestations, which might affect the TMSs used here as a covariate (table 4). It is hard to control for these effects. Drugs prescribed to treat symptoms once they are present will not influence symptom onset data. We used worst-ever depression and irritability scores when validating the use of HD-CCQ to mitigate against the effects of medication prescribed at certain times.

Previous prospective studies of phenotype in HD such as Neurobological Predictors of Huntington's Disease (PREDICT-HD) and TRACK-HD (an observational study of pre-manifest and early stage HD) have shown subtle early reductions in psychiatric and cognitive function years in advance of clinical onset.^7,8^ The HD-CCQ accesses retrospective data from large existing populations of patients with manifest HD and shows similar trends. Because the HD-CCQ is part of ongoing global longitudinal observational studies such as ENROLL-HD, future analyses of larger populations will be possible and of benefit. The presence of psychiatric and cognitive symptoms in HD gene carriers is associated with significantly reduced functional capacity, emphasizing the importance of early recognition and management of these symptoms.^8,45^ Although recent models of HD staging and progression do not directly include psychiatric and cognitive symptoms,^46-48^ work is underway to include them in ongoing observational studies and clinical trials to improve the accuracy of clinical outcome measures.

## Glossary

CI: confidence interval
EHDN: European Huntington's Disease Network
HADS: Hospital Anxiety/Depression Scale
HD: Huntington disease
HDCCQ: HD Clinical Characteristics Questionnaire
ICD-10: *International Classification of Disease, 10th revision*
OR: odds ratio
PBA-s: short form of the Problem Behaviours Assessment
PREDICT-HD: Neurobiological Predictors of Huntington's Disease
REGISTRY: An Observational Study of the European Huntington's Disease Network
SDMT: Symbol-Digit Modalities Test
SIS: Snaith Irritability Scale
TFC: total functional capacity
TMS: total motor score
UHDRS: Unified Huntington's Disease Rating Scale
